# Impact of the 2024 Noto Peninsula Earthquake on Nutritional Status in Residents of an Integrated Medical and Long-Term Care Facility: A Descriptive Study

**DOI:** 10.3390/nu17030506

**Published:** 2025-01-30

**Authors:** Yoji Kokura

**Affiliations:** Department of Nutritional Management, Keiju Hatogaoka Integrated Facility for Medical and Long-Term Care, Hosu 9270023, Japan; yojikokura@hotmail.com; Tel.: +81-0767-53-3335

**Keywords:** nutritional status, nutrition disorders, residential facilities, nursing homes, quadriceps muscle, ultrasonography

## Abstract

Background/Objectives: The dietary changes experienced by residents in long-term care facilities (LTCFs) following an earthquake are poorly understood. This study aimed to examine variations in nutritional status among residents of an Integrated Facility for Medical and Long-term Care (IFMLC), a particular type of Japanese LTCF, after the 2024 Noto Peninsula Earthquake. Methods: This descriptive study was conducted at the single IFMLC. A total of 115 residents living at the facility on 1 January 2024, at the time of the earthquake, were recruited for the study. The focus was the body weight and skeletal muscle mass changes observed before and after the earthquake. The observation period lasted for three months following the earthquake. Results: Water outage persisted for over a month, making dishwashing impossible and leading to an extended reliance on disposable dishes with limited capacity. This situation consequently reduced the variety and volume of meal options and overall energy intake meals. Residents’ body weight significantly decreased 3 months after the earthquake, and the prevalence of weight loss and skeletal muscle mass loss was particularly high in residents with normal swallowing function. To address nutritional deficiencies post-earthquake, the registered dietitian enhanced energy sufficiency through food fortification, oral nutritional supplements, and pre-prepared ready-to-hang liquid formulas. Conclusions: To prevent further weight and skeletal muscle mass reduction among IFMLC residents, providing ample water, and a disaster manual that can be used even with limited resources is essential. Furthermore, preparing for disasters by stockpiling foods and implementing strategies to enhance energy sufficiency is crucial.

## 1. Introduction

Earthquakes, which cause deaths, injuries, destruction of habitats, and disruption of basic needs such as food and water, have a significant impact on nutritional status. Earthquakes are among the natural disasters that frequently occur in many countries. A recent systematic review and meta-analysis reported that the overall prevalence of malnutrition in children after earthquakes was 4.19% for wasting, 16.78% for stunting, 12.59% for underweight, and 28.06% for anemia [1]. In evacuation centers following an earth-quake, several nutritional issues occur, including insufficient intake of energy and protein [2], as well as a lack of vegetables (other than fruits and juices), meat, soy products, dairy, and vitamins [3,4]. Furthermore, a review of factors associated with nutritional problems after natural disasters categorized these issues into four main areas: the size and equipment of emergency shelters, the method of meal provision and meal content in emergency shelters, collaboration with professionals, and cooperation between shelters [5]. Against this background, previous studies have highlighted the importance of nutritional support following a disaster [6,7]. Rapid intervention is particularly necessary for frail, disabled, or geriatric populations [8]. However, these studies focused on evacuation shelters and did not address the nutritional challenges faced by residents in long-term care facilities (LTCFs).

**Figure 1:**
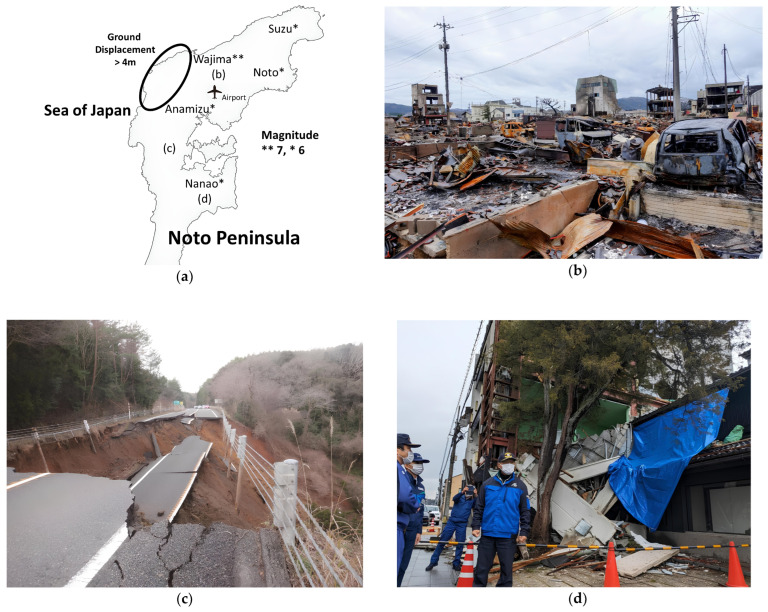
Damage caused by the 2024 Noto Peninsula earthquake in various regions. ** The earthquake had recorded magnitudes of 7; * The earthquake had recorded magnitudes of 6. (**a**) Some parts of the peninsula rose by up to 4 m (13 feet), in response to the uplift, the shoreline advanced by up to 200 m, increasing the total area of the coastal plains by 0.46 km^2^. More than 15 fishing ports in Ishikawa Prefecture reported an uplift. (**b**) In Kawai town, Wajima City, the earthquake sparked a massive blaze that tore through the popular morning market street. The biggest fire was in a populated area with many low-story wooden houses. The burnt area is estimated to be 50,800 m^2^, with about 300 buildings affected. Immediately after the earthquake, the spread of the fire was limited but increased later because the tsunami evacuation detracted from fire-fighting and in any case, enough water could not be secured. (**c**) The Noto Satoyama Kaido, a major road on the Noto Peninsula, collapsed and was closed for >3 months. As of the fourth day after the earthquake, 42 routes and 87 locations were closed. In the Noto Peninsula, where transportation options are limited, supplies and relief efforts delivery faced significant delays immediately following the earthquake. With major roads blocked, transporting essential ingredients for cooking proved difficult. (**d**) In Nanao City, >13,000 buildings were damaged. Wakura Onsen sustained extensive damage. As of 10 February 2024, the area was still without a water supply, and all 22 inns, which together offer approximately 1300 guest rooms and can accommodate around 6600 people, remained closed.

On 1 January 2024, a moment magnitude 7.6 earthquake occurred in the northern Noto Peninsula, Japan. The strong ground motion, tsunami, and massive blaze associated with the earthquake caused severe damage to buildings and infrastructure, resulting in at least 489 casualties in the affected areas [9,10,11,12,13,14] (Table 1, Figure 1). The surface moved upward by as much as 4 m (13 feet) on some parts of the north coast of the Noto Peninsula [11,12,13,14] (Figure 1). This earthquake has brought renewed attention to the critical challenges of managing disaster relief efforts in rapidly aging populations [15]. The northern part of the Noto Peninsula, which suffered the most damage from the earthquake, had a large geriatric population, with 49.0–53.2% of residents aged ≥ 65 years before the earthquake [16]. Compared with the general population, healthy geriatric individuals exhibit increased vulnerability during natural disasters, putting them at risk of deteriorating below the level of physical and cognitive abilities necessary for safe and independent living [17]. Furthermore, the geriatric population faces significantly higher mortality risks after disasters [18,19,20]. In older adults, non-volitional weight loss [21,22] and decreased appendicular skeletal muscle mass index [23] are associated with greater mortality. Individuals aged > 65 years are three times more likely to sustain injuries than younger individuals [24], and geriatric patients who develop acute renal failure experience particularly high mortality rates [25]. Additionally, death rates for the geriatric population after earthquakes can be up to five times higher than those for the rest of the population [26]. This risk is further exacerbated in disrupted healthcare settings such as hospitals and nursing homes [17].

Changes in the nutritional status of residents of LTCFs after an earthquake remain unclear. The integrated facility for medical and long-term care (IFMLC) is a new Japanese LTCF with three key functions: (i) regular medical management, (ii) end-of-life and terminal care services, and (iii) residential living facilities [27]. As of 2024, the IFMLC comprised 926 facilities totaling 53,183 beds, accommodating many geriatric individuals in need of nursing care during their stay [28]. The prevalence of Global Leadership Initiative on Malnutrition (GLIM)-defined malnutrition was 29% in the IFMLC, which may indicate similar or greater malnutrition levels compared to nursing homes [27]. On the other hand, the prevalence of dysphagia in LTCFs and nursing homes is estimated to range from 12.8% to 52.7% [29,30,31]. It has been reported that 39.2% of patients with dysphagia suffer from malnutrition [32]. Additionally, the prevalence of individuals with both dysphagia and malnutrition is approximately 3–29% [33]. Therefore, a close relationship exists between dysphagia and malnutrition [34]. There is a particular concern that geriatric residents with dysphagia in LTCFs may experience a decline in their nutrition.

Therefore, this study aimed to descriptively examine changes in body weight and skeletal muscle mass among IFMLC residents following the 2024 Noto Peninsula earthquake.

## 2. Materials and Methods

### 2.1. Study Design and Participants

This single-center descriptive study was conducted at the Keiju Hatogaoka IFMLC, Japan, which offers rehabilitation services covered by the Japanese insurance system [27]. A total of 115 residents who were already residing in the IFMLC on 1 January 2024, at the time of the earthquake, were recruited for the study. Clinical data, including medical records, were retrospectively collected using the IFMLC’s electronic resident record system. Because regular evaluations were performed at the study facility, the most recent data available at registration were collected as baseline data. The observation period lasted 3 months after the earthquake. Residents who died during the study period, those who were hospitalized, and those with missing data were excluded.

### 2.2. IFMLC

IFMLCs provide comprehensive medical and nursing care management based on service plans customized to each facility [27]. Following physician consultation, these facilities offer a broad spectrum of treatments, including medication and diagnostic tests. IFMLCs also provide end-of-life care and are equipped with diagnostic and therapeutic amenities such as clinical labs, X-rays, and computed tomography imaging. They specialize in various medical services, including nutritional management, respiratory care, and wound treatment, setting them apart from other LTCFs. At IFMLCs, a multidisciplinary team comprising physicians, nurses, pharmacists, therapists, care managers, and dietitians delivers holistic care [27]. Rehabilitation services are a key component, with tailored programs focusing on physical and mental health improvements, provided by dedicated physical and occupational therapists. This personalized approach extends to nutritional care, managed by a full-time registered dietitian who meticulously addresses residents’ dietary needs through comprehensive nutrition care plans.

### 2.3. Timeline of Medical, Public Service, and Nutritional Issues in IFMLC

**Figure 2:**
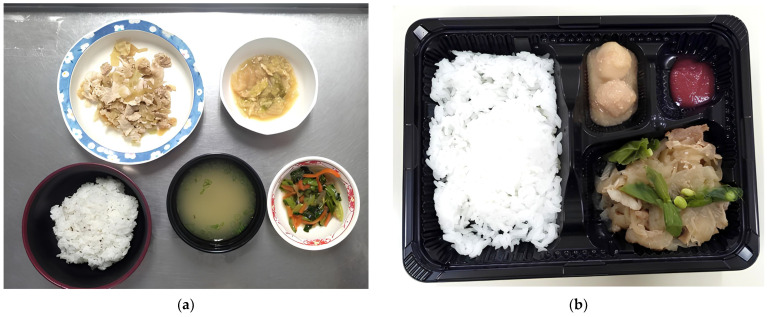
Comparison of meals provided to IFMLC residents before and after the earthquake: (**a**) Meal before the earthquake: The tableware is made of melamine with a large capacity and contains five items: a staple food, a main dish, a side dish, and soup; (**b**) Meal after the earthquake: The tableware is a small, disposable lunch box and contains four items: a staple food, a main dish, and a side dish. IFMLC, integrated facility for medical and long-term care.

Table 2 illustrates the chronology of medical, public service, and nutritional issues that occurred over 3 months at the Keiju Hatogaoka IFMLC following the 2024 Noto Pen-insula earthquake. The water supply was completely disrupted for over a month, severely affecting daily operations and living conditions. Both cooking and facility staff numbers remained insufficient throughout the period, exacerbating the strain on healthcare and nutritional support. Nutrition-related issues evolved over time, with initial difficulties in food delivery transitioning to challenges in ensuring adequate energy intake and meal preparation. Figure 2 illustrates the transition in meal provision during the resource-limited period. The meal on the left represents a standard hospital meal, comprising multiple components such as rice, miso soup, a protein-based main dish, and side dishes with vegetables, designed to meet patients’ nutritional needs, whereas the meal on the right depicts the simplified meal provided during the crisis, which consisted of fewer components: rice or porridge, a single protein source, and limited accompaniments. Energy sufficiency remained below optimal levels for an extended period, starting at only 60% adequacy in January and gradually improving to 90% by March. To address nutritional deficiencies post-earthquake, the registered dietitian enhanced energy sufficiency through food fortification [35], adding protein and medium chain triglyceride oil to staples like porridge, and integrating oral nutritional supplements into meals to replenish protein, vitamins, and trace elements. Additionally, the used of pre-prepared ready-to-hang liquid formulas continued, allowing for nutritional intake during the water outage without the need for washing bottles. The amount of enteral nutrition administered remained nearly the same as before the earthquake.

### 2.4. Data Collection

The pre-earthquake baseline clinical data were obtained during routine assessments. These included age, sex, nursing care level [36], duration of stay at the IFMLC, primary diseases leading to IFMLC admission, comorbidity severity (using the Charlson Comorbidity Index) [37], dysphagia (assessed using the Food Intake LEVEL Scale [FILS]) [38], activities of daily living (measured using the Barthel Index [39]), and undernutrition (evaluated using the GLIM criteria [40]). A registered dietitian assessed the swallowing ability of the residents using the FILS. Based on the swallowing condition, FILS scores were subjectively assessed on a scale ranging from level 1 to level 10, with higher levels indicating greater swallowing ability. Levels 1–3, 4–6, and 7–9 correspond to “severe (complete enteral nutrition)”, “moderate (oral intake with enteral nutrition)”, “mild (oral intake without enteral nutrition)” dysphagia, respectively, whereas level 10 corresponds to “normal” swallowing function. The chronology of medical, public service, and nutritional issues as well as meals provided to residents after the earthquake were collected as post-earthquake data.

### 2.5. Outcome Measurement

The primary outcome was the weight change before and after the earthquake. Weight was measured by nursing staff and caregivers using calibrated scales that allowed measurements while patients remained seated in wheelchairs during routine monthly assessments.

The secondary outcome was the change in skeletal muscle mass before and after the earthquake. Muscle mass in the rectus femoris and vastus intermedius was assessed through ultrasound to determine quadriceps muscle thickness (QMT). QMT was measured at the anterior mid-thigh (midway between the femoral lateral epicondyle and the femoral greater trochanter) [41] in both limbs using B-mode ultrasound (Miruco; Nihon Sigmax Corporation, Tokyo, Japan) with a 10 MHz linear array probe. The ultrasound settings were consistently set to B-mode, dynamic range, gain (dB), and a standardized measurement depth of 6 cm. The findings were correlated with muscle size, validating the use of ultrasound as a reliable method for such assessments, supported by studies using magnetic resonance imaging [42] and dual-energy X-ray absorptiometry [43]. The anterior QMT served as the index for muscle size. All images were analyzed using ImageJ software (version 2.3.0) (National Institutes of Health, Bethesda, MD, USA). At the IFMLC, QMT was being measured using ultrasound by a registered dietitian before the earthquake [44]. In our study groups, the methods used to measure the intraclass correlation coefficient (ICC) (1, 1) for QMT demonstrated high reliability, with an ICC value of 0.952 (95% confidence interval [CI]: 0.871–0.987) [45].

### 2.6. Statistical Analyses

Statistical analyses were conducted using JMP 11.2.1 software (SAS Japan, Tokyo, Japan). Continuous and ordinal data are presented as mean ± standard deviation and median [25th, 75th percentiles], respectively. Categorical data are expressed as frequency (percentage). A complete case analysis was used for missing data. To ensure that our sample was representative of the wider population in the facility, we compared the characteristics of the analyzed group with those excluded to check for any significant differences that might affect the generalizability of our findings. A paired *t*-test with Bonferroni correction for measures was conducted to test within-group differences in weight before the earthquake and at 1, 2, and 3 months post-earthquake. A paired *t*-test was also conducted to examine within-group differences in QMT before the earthquake and 3 months afterward. Additionally, participants were stratified by the severity of their dysphagia according to FILS levels. Background characteristics, weight change, and QMT change across these groups were compared using the chi-squared test, Fisher’s exact test, Student’s *t*-test, and Kruskal–Wallis test. A *p*-value of <0.05 was considered statistically significant.

## 3. Results

### 3.1. Participants Background

**Figure 3:**
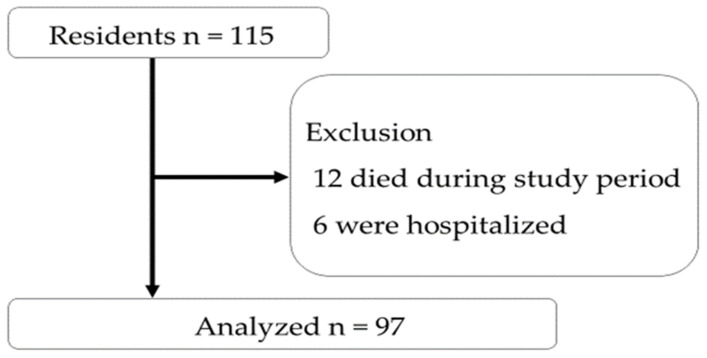
Flowchart of the study participants. The study analyzed 97 participants.

A total of 115 residents were enrolled in this study. Among them, the following were excluded: twelve due to death during the 3-month follow-up and six due to hospitalization before completing the follow-up. Ultimately, 97 residents were included in the study (Figure 3) for analysis of weight changes following the earthquake. A comparison of age, sex, time spent at IFMLC, primary diseases for IFMLC admission, CCI, nursing care level, BI, and FILS between the 15 excluded individuals and the 97 included in the analysis revealed significant differences only in sex, the prevalence of females was 44% in the excluded group, and 72% in the included group (*p* = 0.029).

Table 3 presents the characteristics of the 97 residents who continued to be admitted to the IFMLC after the earthquake. The median age was 89 years (interquartile range [IQR]: 82–93), and 72% were female. The median body mass index (BMI) was 19 kg/m^2^ (IQR: 17–21), and the median duration of stay was 749 days (IQR: 261–1194). Cerebrovascular disease (43%) and dementia (18%) were the most common primary diagnoses. Most participants (64%) had mild dysphagia, requiring oral intake without enteral nutrition, whereas 15% had severe dysphagia, requiring complete enteral nutrition.

### 3.2. Changes in Weight and QMT

**Figure 4:**
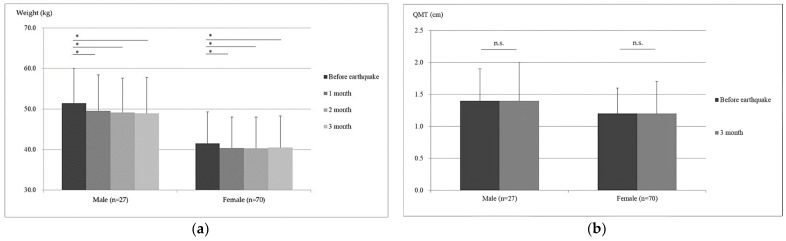
Weight and QMT comparison before and after the earthquake: (**a**) Paired *t*-test with Bonferroni correction. Data points represent the average weight of male and female participants measured at different time points (before and at 1, 2, and 3 months after the earthquake). (**b**) Paired *t*-test. Comparison of QMT, including the rectus femoris and vastus intermedius, in male and female participants before and at 3 months after the earthquake. * *p* < 0.05 vs. weight before the earthquake; n.s., not significant. QMT, quadriceps muscle thickness.

Figure 4 illustrates the changes in weight and QMT for male and female participants over time, focusing on the periods before and after the earthquake. Both male and female residents experienced significant weight loss at each time point post-earthquake (*p* < 0.05). Conversely, no significant changes in QMT were observed over the 3-month period for either male or female participants.

### 3.3. Outcomes by Weight and QMT Change

Table 4 compares the basic characteristics of the participants before the earthquake, weight changes after the earthquake, and QMT among participants categorized by dysphagia severity, as assessed using FILS. The median BMI before the earthquake was significantly lower in participants with severe and mild dysphagia (19 kg/m^2^) compared to those with normal swallowing function (23 kg/m^2^, *p* < 0.05). Energy intake (kcal/IBW/day) before the earthquake was also significantly lower in the severe (19 kcal/IBW/day) and moderate (21 kcal/IBW/day) dysphagia groups compared to the normal swallowing group (27 kcal/IBW/day, *p* < 0.05). The prevalence of GLIM-defined malnutrition was highest in the severe (43%) and moderate (50%) dysphagia groups compared to the normal swallowing group (15%), but this difference was not statistically significant (*p* = 0.321). The normal swallowing group showed maximum weight change (−1.5 kg), whereas no weight loss was observed in the severe dysphagia group (+0.1 kg, *p* = 0.036). A higher percentage of participants in the normal swallowing group experienced weight loss (92%) compared to that in the severe dysphagia group (43%, *p* = 0.021). The mild dysphagia and normal swallowing groups exhibited the highest percentages of QMT decrease, with 63% and 62% of participants showing QMT loss, respectively. Conversely, the severe (7%) and moderate (25%) dysphagia groups exhibited lower proportions of participants with QMT decrease.

## 4. Discussion

We descriptively explored changes in body weight and skeletal muscle mass among IFMLC residents following the 2024 Noto Peninsula earthquake using retrospective data. The study revealed that residents’ body weight significantly decreased 3 months after the earthquake and that the prevalence of weight loss and skeletal muscle mass loss was particularly high in the normal swallowing group. To the best of our knowledge, this study is the first to investigate changes in body weight and skeletal muscle mass among residents in an IFMLC after a major earthquake.

The weight of IFMLC residents decreased over the 3 month-period following the earthquake, and the prevalence of weight loss and skeletal muscle mass loss was particularly high in the normal swallowing group. Previous studies have provided inconsistent results regarding changes in weight among victims after an earthquake. In 2014, Inoue et al. [46] reported that among 236 disaster evacuees aged 9–88 years (mean age 52 years), 23% experienced weight loss and 28% reported decreased food intake 1 month after the Great East Japan Earthquake. The weight loss was attributed to the consumption of imbalanced diets, which may have caused more gastrointestinal symptoms for the survivors [38]. In 2016, Ohira et al. [47] reported that among evacuees (mean age 66–67 years) following the Great East Japan Earthquake, weight significantly increased by 1.6 years after the disaster for both evacuees (*n* = 9671) and non-evacuees (*n* = 17,815). The weight gain was more pronounced in evacuees than in non-evacuees (+1.2 kg vs. +0.3 kg, *p* < 0.001), and evacuation was associated with an increased risk of becoming overweight [47]. However, the participants in these prior studies were not residents of LTCFs, and the observation periods varied. Consequently, it is premature to draw conclusions about weight changes in residents of LTCFs following an earthquake. In the present study, the residents’ weight loss was attributed to a reduction in the energy content of meals provided at IFMLC, with the percentage of energy sufficiency declining to 60%. This study’s results are valid because it has been reported that insufficient energy intake decreases QMT in geriatric individuals [48].

Notably, the largest decreases in body weight and skeletal muscle mass were observed in the normal swallowing group. The reason for this result is related to the BMI, energy intake, and ADL scores of the normal swallowing function group before the earthquake. This group had the highest measurements among the four groups, with a BMI of 23 kg/m^2^, an energy intake of 27 kcal/IBW/day, and an ADL score of 70 points. Being well-built, well-nutritional intake, and active before the earthquake, the normal swallowing group was most affected by the post-disaster reduction in energy intake. Consequently, they exhibited the greatest decrease in body weight and skeletal muscle mass. In contrast, it is speculated that the severe, moderate, and mild groups, which had lower energy intake, BMI, and activity levels before the earthquake, were less affected by the reduction in energy intake following the earthquake. Weight loss over one year is an independent and significant risk factor for 6-month mortality among nursing home residents aged 65 and older [49]. Additionally, decreased skeletal muscle mass is linked to the survival rates of institutionalized geriatric nursing home residents [50]. Therefore, after an earthquake at IFMLC, special attention must be paid to the decrease in body weight and skeletal muscle mass in the normal swallowing group.

The decrease in food energy provision after the earthquake was due to a combination of factors, including the disruption of daily life infrastructure and a shortage of facility staff. Among the various types of daily life infrastructure, the interruption of the water supply was a particularly significant issue. These results provide new insights into the factors influencing both the quantity and quality of food provided after an earthquake. In 2014, Tsuboyama-Kasaoka et al. [51] reported that when the gas supply was available, the twice-daily provision of “balanced” meals (containing grains, vegetables, and meat/fish) was more frequent than when there was no gas supply in emergency shelters during the Great East Japan Earthquake of 2011. However, the availability of water and electricity supplies did not significantly affect the provision of balanced meals [41]. In the present study, even though the gas supply was restored approximately 10 days after the earthquake, the percentage of energy sufficiency continued to decline. This decline was attributed to the delayed restoration of the water supply. Nojima et al. [52] reported that water supply restoration reached nearly full completion 1 month after the 2016 Kumamoto Earthquake, with over 80% restored within a month following both the 2011 Great East Japan Earthquake and the 1995 Great Hanshin-Awaji Earthquake. Conversely, the restoration process after the 2024 Noto Peninsula Earthquake has been slower due to severe damage to water pipes, purification plants, and distribution ponds [52]. The water outage, which lasted for more than a month, made it impossible to wash dishes, necessitating the prolonged use of disposable dishes with limited capacity. This, in turn, led to a reduction in the variety and quantity of meal items, as well as the overall energy provided in the meals.

This result underscores the importance of a reliable water supply in maintaining adequate food provision after an earthquake. Additionally, if the number of cooking staff, nurses, and caregivers had remained the same as before the earthquake, it might have been possible to increase the number of meal containers and maintain the energy content of meals despite the smaller dish capacity. However, these facility staff members were also affected by the earthquake-induced fire, tsunami, and destruction of their homes, which prevented them from coming to work. Consequently, the combination of the water outage and labor shortage led to a reduced energy supply. To mitigate such issues in future disasters, facilities should always have disposable dishes with sufficient capacity on hand. Disaster response manuals should be designed to remain operable even with limited manpower. More importantly, during this period, the registered dietitian employed several strategies to increase energy sufficiency, including food fortification, oral nutritional supplements, and enteral nutrition. Food fortification is a common practice to improve the nutrient status in a food product that helps people combat deficiency symptoms for various ailments [35]. This process uses macronutrients or micronutrients in the food to enhance the nutritional status. After the earthquake, to address initial nutritional deficiencies, the registered dietitian enhanced the energy and protein content of staple foods like porridge by adding protein powder and medium chain triglyceride oil. Additionally, Oral Nutrition Supplementation was incorporated into residents’ lunches to compensate for broader nutrient deficiencies, including protein, vitamins, and trace elements. Furthermore, the use of ready-to-hang liquid formulas, which had been utilized before the earthquake, continued as part of the enteral nutrition regimen. Ready-to-hang liquid formulas were particularly valuable during the water outage as they eliminated the need to wash nutrition bottles. Particularly, residents in the severe dysphagia group (who require complete enteral nutrition) and used ready-to-hang liquid formulas did not experience any loss in body weight or skeletal muscle mass. Therefore, these formulas might be effective in maintaining the nutritional status of residents in long-term care facilities during disasters.

Loss of weight and skeletal muscle mass is associated with poor outcomes in residents of LTCFs. Non-volitional weight loss and reduced muscle mass are included in the phenotypic criteria of the GLIM framework, a recently proposed global diagnostic method for malnutrition [32]. Consequently, non-volitional weight loss and reduced muscle mass increase the risk of malnutrition. Malnutrition diagnosed using the GLIM criteria in an IFMLC has been identified as a risk factor for 1-year all-cause mortality [53]. Furthermore, reduced skeletal muscle mass has been associated with a decline in activities of daily living [44,45]. Therefore, post-earthquake LTCFs must prioritize minimizing the loss of body weight and skeletal muscle mass to prevent deterioration in both mortality rates and physical function outcomes for residents.

Some limitations of this study should be acknowledged. First, it did not examine changes in daily activity levels or the implementation of oral care for residents before and after the earthquake. Daily activity levels and oral care practices could have influenced the dietary intake of IFMLC residents and, consequently, the loss of body weight and skeletal muscle mass. Second, the outcomes of this descriptive study may not be as broadly applicable because it was restricted to a single IFMLC. In the present study, a comparison of sex between the excluded subjects and those included in the analysis revealed significant sex differences. Additionally, the basic characteristics of residents at our facility differ from those typically observed in average IFMLCs facilities across Japan [54]. For instance, the combined proportion of residents with nursing care levels 4 and 5 was over 80% nationwide, compared to 56% in this study. Furthermore, the median BI nationwide was 5, while in this study, it was higher at 15. Therefore, more research is needed to determine whether the results of this study apply to different IFMLCs. Finally, the extent of damage to essential services such as electricity, water, and gas supply after the earthquake varies greatly between countries and regions, depending on the level of relief efforts and the support systems provided by local governments. This variation affects the external validity of the study’s findings. Despite these limitations, this study highlights the previously overlooked issue of changes in the nutritional status of LTCF residents following an earthquake.

## 5. Conclusions

This study demonstrated that residents’ body weight significantly decreased 3 months after the earthquake and that the prevalence of weight loss and skeletal muscle mass loss was particularly high in the normal swallowing group. Among IFMLC residents, ensuring an adequate water supply, having disposable dishes with sufficient capacity, and developing a disaster manual operable even with limited manpower are essential to prevent weight and skeletal muscle mass loss after an earthquake. More importantly, in preparation for disasters, it is crucial to stockpile foods and implement strategies to enhance energy sufficiency. These strategies include practicing food modification, utilizing oral nutritional supplements, and employing ready-to-hang liquid formulas. Registered dietitians could recommend that facility managers keep supplies of protein powders, medium-chain fatty acid oils, oral nutritional supplements, and ready-to-hang liquid formulas. These recommendations can also inform the development of practical guidelines for improving nutrition during emergencies.

## Figures and Tables

**Table 1:** Damage statistics from the 2024 Noto Peninsula Earthquake.

Date and Time (Japan Standard Time)	1 January 2024 (16:10)
Magnitude	7.6
Human damage	
Deaths *	489
Missing	2
Injured	1379
Building damage	
Collapsed (households)	6445
Partially collapsed (households)	23,225
Minor damage	120,029

* Number of deaths includes those caused by indirect factors related to the earthquake.

**Table 2:** Chronology of medical, public service, and nutritional issues occurring in the integrated medical and long-term care facility after the 2024 Noto Peninsula earthquake.

	January						February			March		
	Day 1	Day 2	Day 3	Days 4–10	Days 11–20	Days 21–31	Days 1–10	Days 11–20	Days 21–28	Days 1–10	Days 11–20	Days 21–31
Utilities												
Electric power supply	×	×	✓	✓	✓	✓	✓	✓	✓	✓	✓	✓
Water supply	×	×	×	×	×	×	✓	✓	✓	✓	✓	✓
Gas supply	×	×	×	×	✓	✓	✓	✓	✓	✓	✓	✓
Healthcare issues	Hypothermia, endogenous diseases, burns, psychiatric shock, and lack of medication for preexisting conditions			Gastritis, pressure ulcers, exacerbation of chronic conditions, respiratory diseases, and infectious diseases			Decline in oral function and activities of daily living, musculoskeletal diseases, deep venous thrombosis, and digestive disorders (diarrhea, constipation)					
	Noncommunicable diseases (hypertension, diabetes mellitus, chronic renal failure, etc.), oxygen-dependent management, insomnia, and skin-related disorders											
Number of registered dietitians/regular staff	0/1	0/1	1/1	1/1	1/1	1/1	1/1	1/1	1/1	1/1	1/1	1/1
Number of cooking staff/regular staff	2/8	2/8	2/8	4/8	4/8	4/8	6/8	6/8	6/8	6/8	6/8	8/8
Number of facility staff/regular staff	30/60	30/60	30/60	44/60	44/60	44/60	50/60	50/60	50/60	55/60	55/60	55/60
Nutrition issues	Delivery of food ingredients disrupted due to worsening road conditions; shortage of drinking water			Inability to wash dishes, necessitating the use of smaller-capacity disposable dishes; insufficient energy provided to residents			Reduced medical and nursing staff leading to inadequate meal assistance; insufficient oral care provided to residents					
Percentage of energy sufficiency (%)												
Oral intake	60	60	60	65	65	70	80	80	85	85	90	90
Enteral nutrition	90	90	90	100	100	100	100	100	100	100	100	100

✓ Service/resource was available; × Service/resource was unavailable.

**Table 3:** Characteristics of IFMLC residents after the earthquake.

Characteristics	All (*n* = 97)
Age (years), median (IQR)	89 (82–93)
Sex (female), *n* (%)	70 (72)
BMI, (kg/m^2^), median (IQR)	19 (17–21)
Time spent at IFMLC, (days), median (IQR)	749 (261–1194)
Primary diseases for IFMLC admission, *n* (%)	
Cerebrovascular disease	42 (43)
Dementia	17 (18)
Orthopedic diseases	11 (11)
Heart failure	7 (7)
Lung disease	2 (2)
Disuse syndrome	4 (4)
Other diseases	14 (15)
CCI, (points), median (IQR)	2 (1–3)
Nursing care level, *n* (%)	
1	10 (10)
2	13 (13)
3	20 (21)
4	20 (21)
5	34 (35)
Dysphagia severity by FILS, *n* (%)	
Severe (complete enteral nutrition)	14 (15)
Moderate (oral intake with enteral nutrition)	8 (8)
Mild (oral intake without enteral nutrition)	62 (64)
Normal swallowing function	13 (13)
Barthel index	15 (0–48)

IQR, interquartile range; BMI, body mass index; IFMLC, integrated facility for medical and long-term care; CCI, Charlson Comorbidity Index; FILS, Food Intake LEVEL Scale.

**Table 4:** Comparison of weight and QMT change disaggregated by dysphagia severity.

	Dysphagia Severity by FILS				
Characteristics	Severe (Complete Enteral Nutrition)	Moderate (Oral Intake with Enteral Nutrition)	Mild (Oral Intake Without Enteral Nutrition)	Normal Swallowing Function	*p*-Value
Number of individuals, *n* (%)	14 (15)	8 (8)	62 (65)	13 (13)	
Basic information before the earthquake					
Age (years), median (IQR)	85 (80–94)	89 (76–91)	91 (82–95)	87 (82–90)	0.451 ^(a)^
Sex (female), *n* (%)	11 (79)	5 (63)	46 (74)	8 (62)	0.646 ^(b)^
BMI, (kg/m^2^), median (IQR)	19 (17–21) ^(c)^*	18 (15–22)	19 (17–21) ^(c)^*	23 (20–25)	0.004 ^(a)^
Energy intake, (kcal/IBW/day), median (IQR)	19 (18–21) ^(c)^**	21 (18–26) ^(c)^*	25 (22–28)	27 (26–28)	<0.001 ^(a)^
Barthel index	0 (0–0) ^(c)^**	10 (0–41) ^(c)^*	18 (0–40) ^(c)^*	70 (48–78)	<0.001 ^(a)^
GLIM-defined malnutrition, *n* (%)	6 (43)	4 (50)	20 (32)	2 (15)	0.321 ^(b)^
Weight (kg)					
Before earthquake	43 (35–47) ^(c)^**	44 (33–52)	42 (36–48) ^(c)^**	56 (48–62)	0.003 ^(a)^
Change	0.1 (−1.3–2.3) ^(c)^*	−0.9 (−2.9–1.9)	−1.0 (−2.8–0.1)	−1.5 (−1.5–−0.6)	0.036 ^(a)^
Decrease, *n* (%)	6 (43)	4 (50)	45 (73)	12 (92)	0.021 ^(b)^
QMT					
Before earthquake	0.99 (0.76–1.48) ^(c)^*	1.32 (0.94–1.66)	1.08 (0.85–1.39) ^(c)^**	1.64 (1.33–2.22)	0.004 ^(a)^
Change	0.17 (0.04–0.52) ^(c)^*	0.01 (−0.05–0.21)	−0.06 (−0.14–0.07)	−0.05 (−0.18–0.16)	0.002 ^(a)^
Decrease, *n* (%)	1 (7) ^(c)^*	2 (25)	39 (63)	8 (62)	<0.001 ^(b)^

^(a)^ Mann–Whitney U test, ^(b)^ Fisher’s exact test, and ^(c)^ Significant differences compared with the normal group by Steel–Dwass test; * *p* < 0.05, ** *p* < 0.01. FILS, Food Intake LEVEL Scale; IQR, interquartile range; BMI, body mass index; GLIM, Global Leadership Initiative on Malnutrition; IBW, ideal body weight; QMT, quadriceps muscle thickness.

## Data Availability

Data are contained within the article.

## Abbreviations

The following abbreviations are used in this manuscript:

BMI	Body Mass Index
CCI	Charlson Comorbidity Index
FILS	Food Intake LEVEL Scale
GLIM	Global Leadership Initiative on Malnutrition
IBW	Ideal body weight
ICC	Intraclass correlation coefficient
IFMLC	Integrated facility for medical and long-term care
IQR	Interquartile range
LTCM	Long-term care facility
QMT	Quadriceps muscle thickness
